# From ATP synthase dimers to C-ring conformational changes: unified model of the mitochondrial permeability transition pore

**DOI:** 10.1038/s41419-017-0042-3

**Published:** 2017-12-04

**Authors:** Giuseppe Federico Amodeo, Maria E. Solesio, Evgeny V. Pavlov

**Affiliations:** Department of Basic Sciences, New York University, College of Dentistry, 345 East 24th Street, New York, NY 10010 USA

Mitochondrial permeability transition (mPT) is a phenomenon of sudden increase of the permeability of the mitochondrial inner membrane in response to excessive accumulation of calcium and/or oxidative stress. It is generally agreed that mPT is caused by the opening of a large channel (mitochondrial permeability transition pore or mPTP) that allows flux of ions and small molecules up to 1.5 kDa across the inner membrane. Activation of mPT causes mitochondrial membrane depolarization and a loss of mitochondrial function. It is believed that mPT is a central event leading to the mitochondrial dysfunction and is the major cause of cell damage in a wide range of pathologies from acute conditions related to stroke and heart attack to neurodegenerative diseases^1,2^. Currently the molecular composition of mPTP is not entirely understood and the nature of the core “pore” part of mPTP remains subject of hot debates. This significantly limits both our basic understanding of one of the most fundamental steps leading to cell death and the translational use of the mPTP as a drug target for disease treatment.

Several recent independent studies placed mitochondrial ATP synthase (ATPase) as a central candidate for the location of the enigmatic “pore” structure. While all the studies agreed that ATPase is central to the mPTP, some of them proposed the C-subunit ring of ATPase as the central protagonist of the “pore” formation^3–5^ while others pointed out the essential role of ATPase dimers. The recent study by Bonora and colleagues^6^ published in EMBO Reports provides experimental evidence that offers a resolution to these seemingly controversial points of view. Based on an extensive set of experiments authors propose the mechanism according to which mPTP activation likely occurs as a two-step process: the dissociation of ATPase dimers followed by the rearrangement of the C-ring of the ATPase monomers.

In the previous studies this group demonstrated a critical role for the C-subunit of ATPase in the mPTP^7^. Here, they expand their earlier studies in an attempt to further elucidate how the C-subunit participates in the process of mPTP activation and how this could be linked to the more global reorganization of the ATPase complex. First, they use isolated rat liver mitochondria and live-cell culture models of mPTP to establish that activation of the mPTP leads to the dissociation of dimers. In both cases they detected significant increase in the monomeric forms of ATPase, an effect, which was inhibited by CsA suggesting a specific link to the mPTP. Next, they investigated the relationship between stabilization of the ATPase dimers and mPTP activity. To do this they used siRNA against ATPIF1 factor, which is known to promote dimerization. They confirm that the absence of ATPIF1 makes cells to more readily undergo mPTP further supporting the idea about the importance of dimers dissociation for mPTP activation. This interpretation was further supported by the observation that depletion of another factor that is involved in dimerization (subunit E of F0 complex (ATP5I)) or overexpression of its inactive mutant leads to the enhancement of the mPTP activity.

After establishing the link between ATPase dimer dissociation and mPTP activation the authors move on test possible changes in C-subunit rings. They find that mPTP activity is modified in mutants which have been predicted to cause deformation in the C-ring structure. Finally, authors performed ex vivo experiments to explore the possibility of ascertain if pharmacological agents that target C-subunit can affect stress induced cell death. Strikingly, they found that in experiments with perfused ischemic isolated hearts use of DCCD—C-subunit rearranging agent led to even better protection against ischemic damage than CsA—specific blocker of mPT.

Taken together, this study provides a valuable insight into the molecular mechanism of mPTP activation. This data suggests that, prior to mPTP activation, dimers of ATPase need to dissociate allowing the formation of mPTP through the rearrangement of the C-subunit ring of the ATPase.

**Figure 1:**
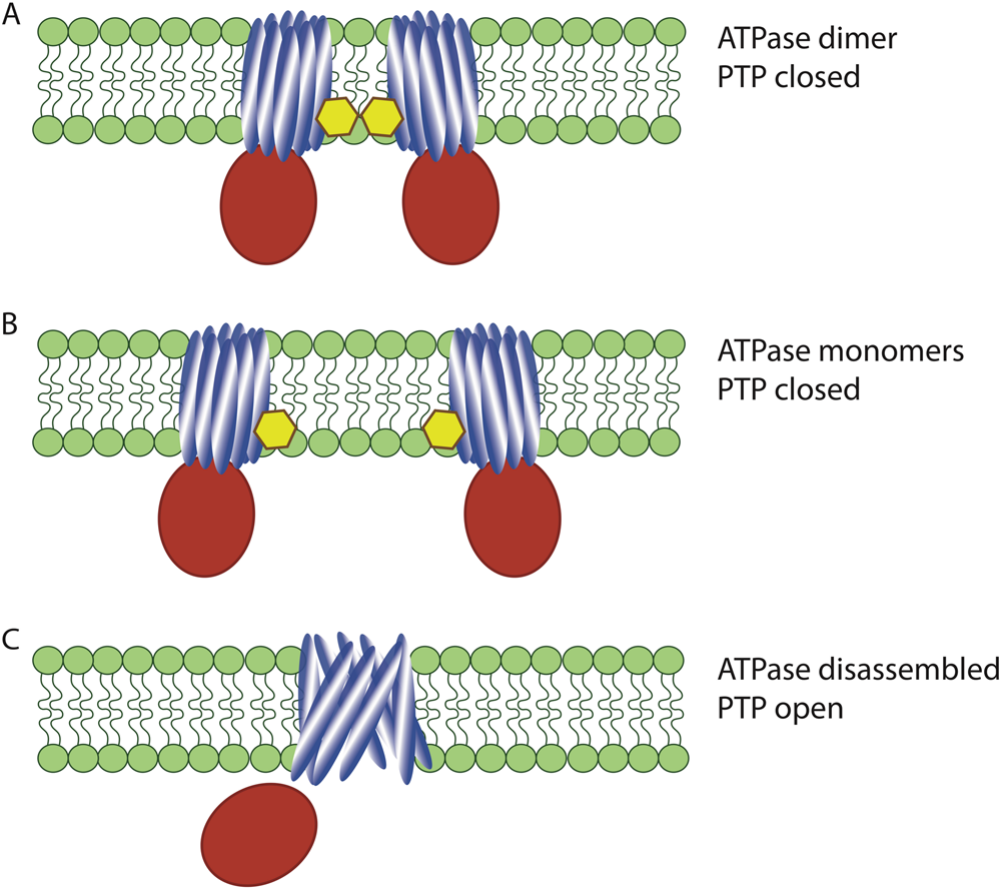
Proposed model for the opening of the mPTP Dissociation of ATP synthase dimers **a** into monomers **b** induced by high level of calcium is followed by the dislocation of thesubunit F1 (red) and conformational rearrangement of the C-ring (blue) **c **allowing the opening ofthe pore

While this study doesnot directly address the issue of the structure of the mPT “pore” it provides explanation to the experimental models of mPTP that involve ATPase dimers and C-subunit ring as important participants in its opening. Indeed, multiple functional experimental data prove that the C-subunit is capable to form large “mPTP-like” pores^5,8^. However, simulation investigations of the complete ATPase complex suggest that it is very unlikely that the C-subunit ring can be converted into the large pore while this complex is intact^9^. This study combined with earlier work of Alavian et al.^5^ suggest the possibility that rearrangements of the C-ring most likely require dimer dissociation (Figure 1b) followed by disruption of F1–F0 interactions. These rearrangements will modify the C-subunit ring organization in such a way that it becomes available for conformational reorganization. This conformational reorganization likely involves polyhydoxybutyrate and inorganic polyphosphates as chaperones capable to induce formation of the large pores^10,11^.

At first appearance this study is in contradiction with previous reports by Giorgio et al.^12^, who argue the dimerization (not dissociation) is critical for mPTP. In fact, this contradiction can be resolved if we consider that mPTP development is likely a very dynamic process. Taking into account that opening of the mPTP requires dramatic conformational changes, it is conceivable that the probability of such change and mPTP opening is increased at the transition moment between dimer and monomer states. Thus, mPTP activity would not correlate directly to the amount of monomers but rather be a function of the probability of dimer-monomer transition, and as such will be increased when the amount of dimers available for transition is increased. This interpretation would satisfy both models.

The exact mechanism of C-subunit ring transition into mPTP remains to be established. Of interest, it should be noted that according to recent data CsA sensitive, calcium-induced membrane permeabilization can be detected even in the absence of C-subunit^9^. This agrees with previous proposed model that in addition of the primary mechanism mPT can occur through alternative pathways that presumably involve misfolded proteins^13^. Taking into account strong similarities in mPT properties of wild-type and C-subunit knock-out cells, it is tantalizing to propose that in wild-type organisms mPTP activation requires unfolding of the native form of the C-subunit and that in the absence of the C-subunit, the mPTP “pore” can be formed by other misfolded membrane protein. With this respect, it is interesting that at least one transporting protein (adenine nucleotide translocator - ANT) can be converted into mPTP-like channel in a CsA-dependent manner^14^. While ANT has been dismissed as a mPTP “pore” candidate^15^ it is conceivable that it could fit into this role in the absence of the primary pathway^13^.

In conclusion, the current study provides critical insights into the roles of ATPase and C-subunit participation in mPTP formation and activation. This work will help to guide future studies of the specific structural steps that are needed for transformation of the intact functional ATPase dimers into nonspecific large pore responsible for mPTP.
